# Supplementary material for: Injectable therapeutic system incorporating neurogenesis-programmed stem cells concomitantly promoting muscle regeneration treats stress urinary incontinence
Source: Nat Commun. 2025 Sep 25;16:8404. doi: 10.1038/s41467-025-63421-2 (PMC12462450; doi:10.1038/s41467-025-63421-2)
Supplement: Supplementary file 1 — Supporting Information [file 41467_2025_63421_MOESM1_ESM.pdf]

Supplementary Information

Injectable therapeutic system incorporating neurogenesis-programmed stem cells concomitantly promoting muscle regeneration treats stress urinary incontinence

*Wenzhuo Fang[#1], Xuan Du[#2], Ranxing Yang[#1], Meng Liu[1], Ming Yang[1], Yangwang Jin[1], Guo Gao[*2], Qiang Fu[*1], Ying Wang[*1]*

[1]Department of Urology, Shanghai Sixth People's Hospital Affiliated to Shanghai Jiao Tong University School of Medicine, Shanghai Jiao Tong University, Shanghai, 200233, P. R. China;
[2]School of Automation and Intelligent Sensing, Shanghai Jiao Tong University, Shanghai, 200240, China.

[*]Corresponding author E-mail:
Ying Wang: sdzbbswangying@alumni.sjtu.edu.cn
Qiang Fu: jamesqfu@126.com
Guo Gao: guogao@sjtu.edu.cn

[#]These authors contributed equally to this work.

This file includes:
Supplementary Fig.1 to 22.
Supplementary Table.1 to 3.

1. Supplementary figures

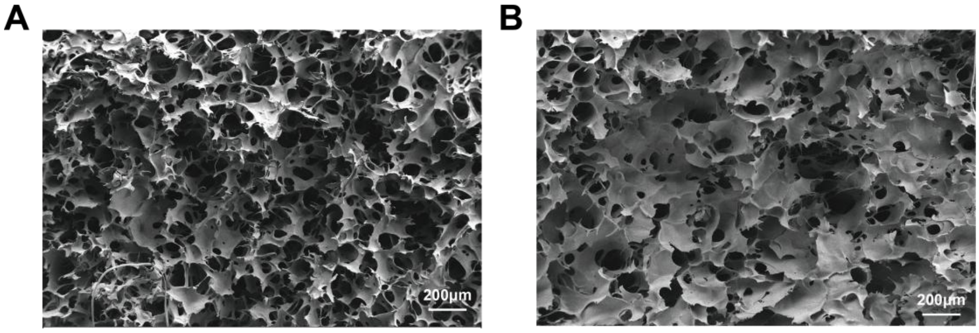

Supplementary Fig. 1 Vertical scanning electron microscopy (SEM) images of (A) PNIPAm-C and (B) PNIPAm-C/Leu/dECM hydrogels (scale bar = 200 μm) (n = 3 independent samples).

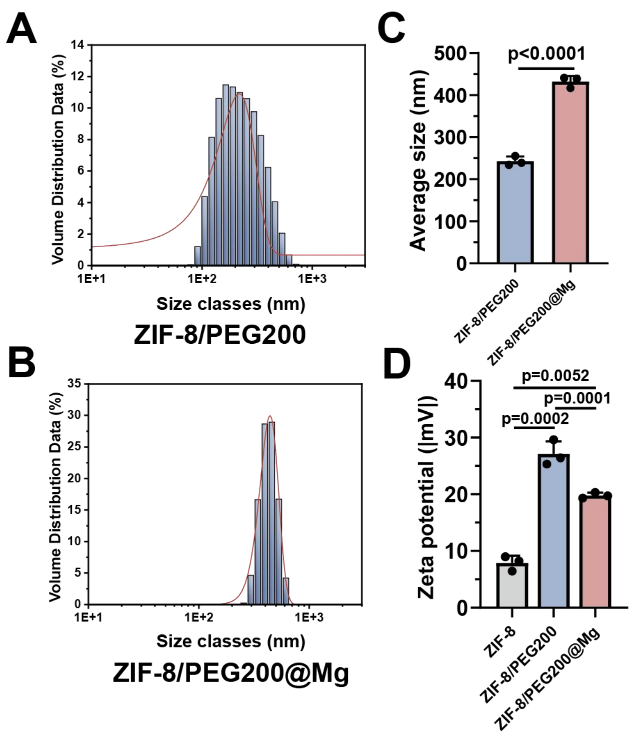

Supplementary Fig. 2 (A-C) DLS and (D) zeta-potential analysis of ZIF-8, ZIF-8/PEG200 and ZIF-8/PEG200@Mg NPs. Data are expressed as the mean ± SD (n = 3 independent samples). p values calculated using one-tailed unpaired t-test. Source data are provided as a Source Data file.

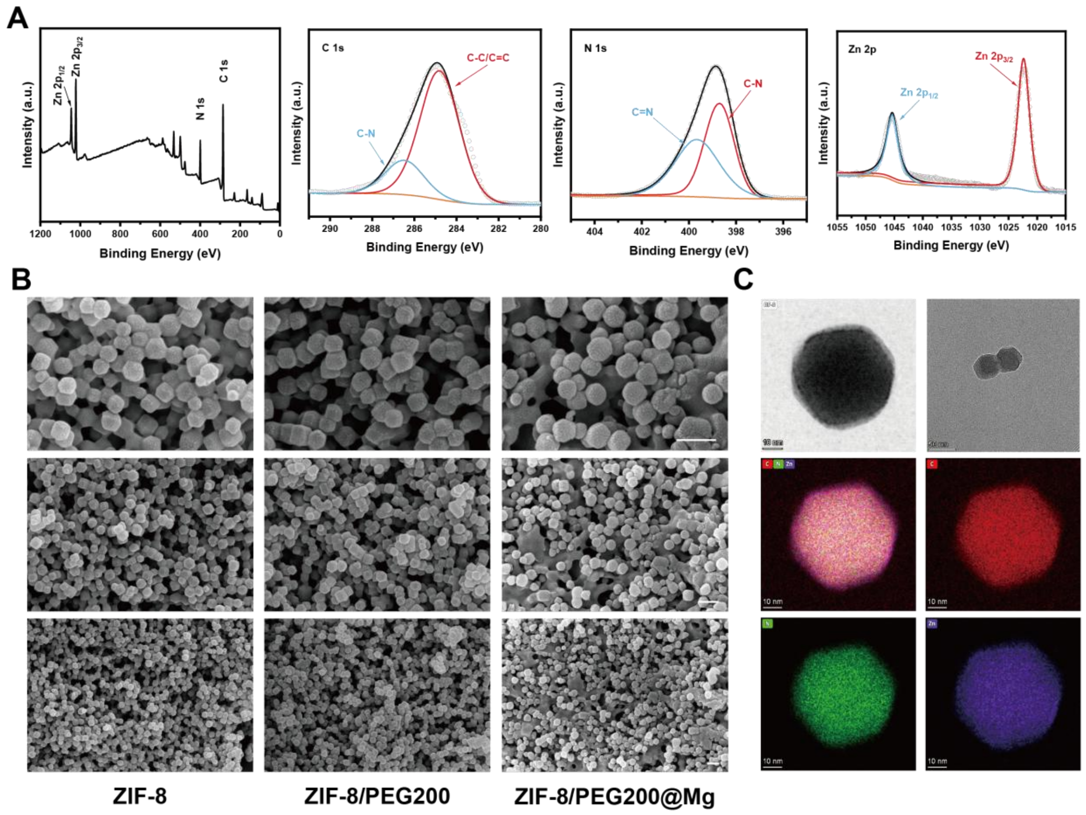

Supplementary Fig. 3 (A) *XPS* spectra of C 1*s*, N 1*s* and Zn 2*p* of ZIF-8. (B) SEM images of ZIF-8, ZIF-8/PEG200 and ZIF-8/PEG200@Mg NPs (scale bar = 200 μm). (C) TEM and EDS elemental mapping of C (red), N (green) and Zn (purple) for ZIF-8. Representative image from n = 3 independent experiments with similar results.

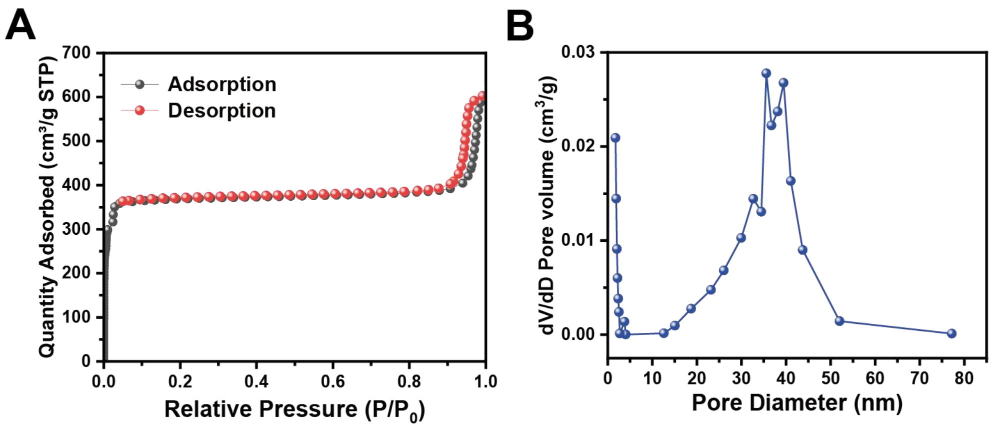

Supplementary Fig. 4 (A) N_2 adsorption/desorption isotherms and (B) pore width distribution of ZIF-8 NPs.

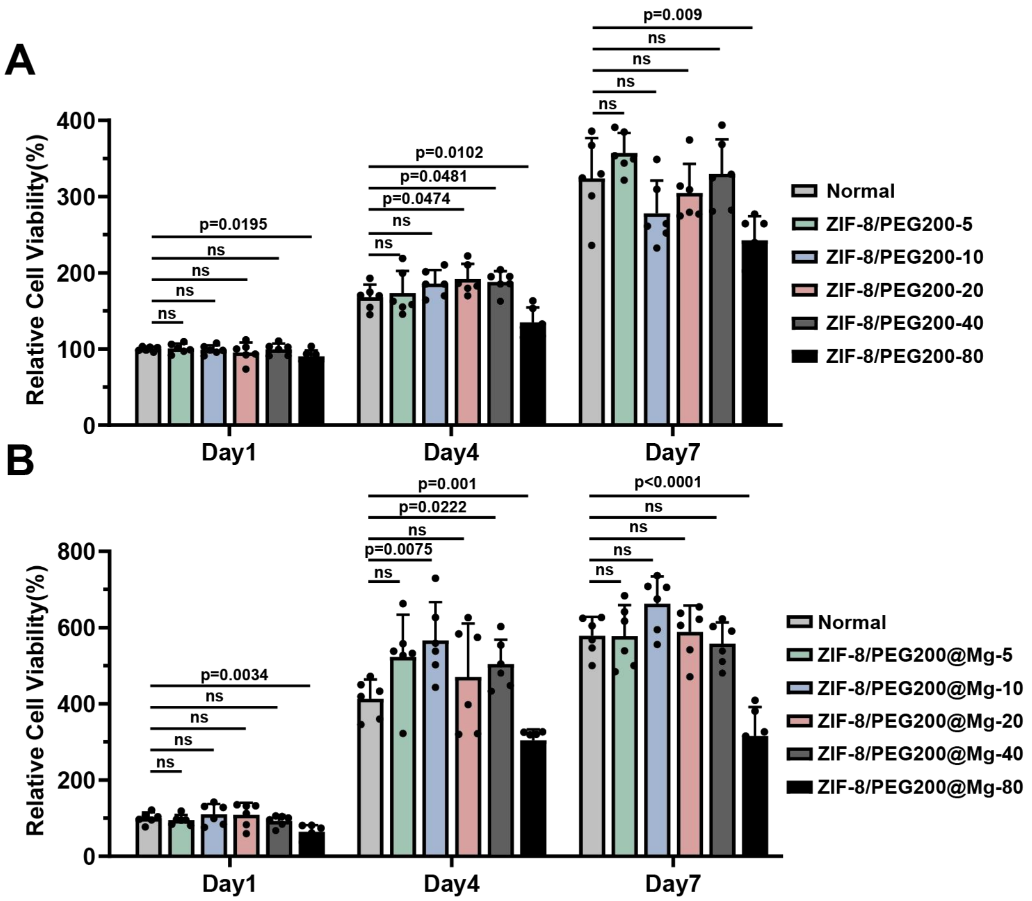

Supplementary Fig. 5 CCK8 assay of ADSCs treated with different concentration of (A) ZIF-8/PEG200 and (B) ZIF-8/PEG200@Mg NPs. Data are expressed as the mean ± SD (n = 6 biologically independent samples). ns, not significant ($p > 0.05$). p values calculated using one-tailed unpaired t-test. Source data are provided as a Source Data file.

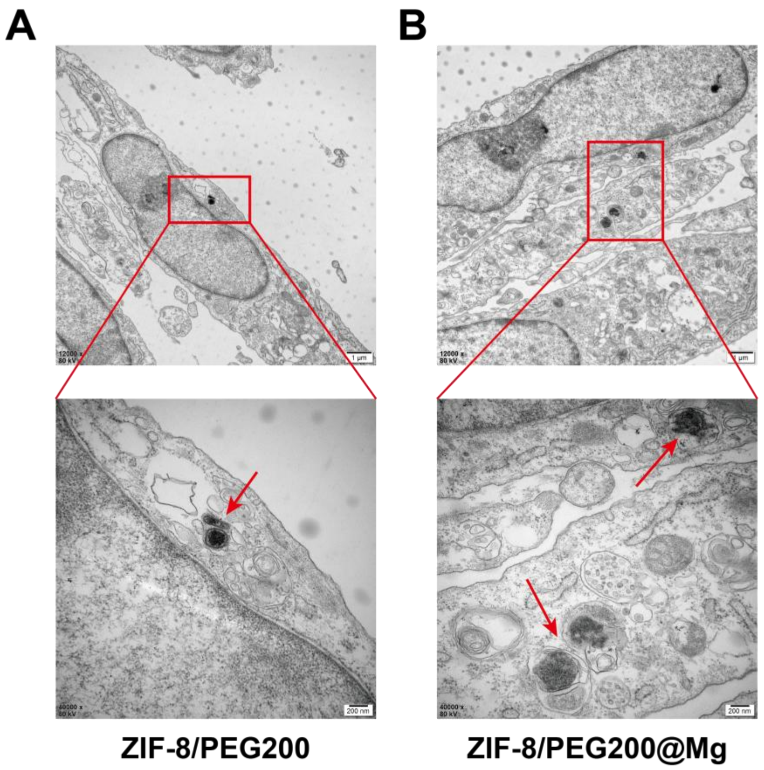

Supplementary Fig. 6 TEM images of ADSCs phagocytosing (A) ZIF-8/PEG200 and (B) ZIF-8/PEG200@Mg NPs. The inset is a magnified image of the area marked by the red rectangle showing the location of MOF NPs (red arrow). Representative image from n = 3 independent experiments with similar results.

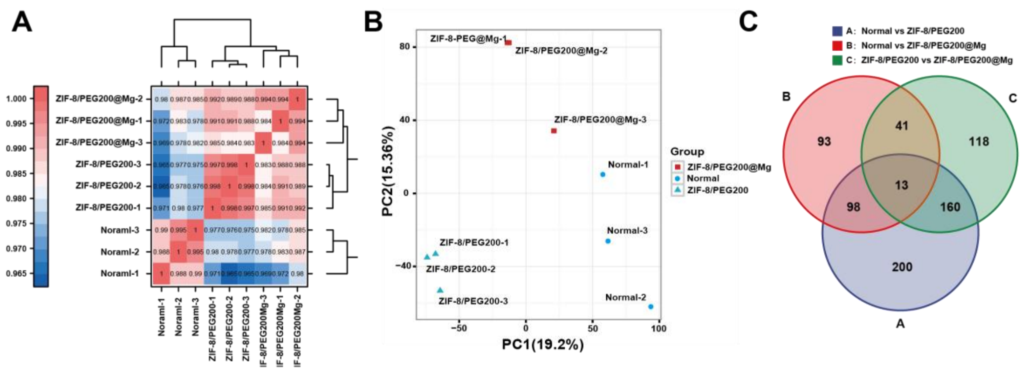

Supplementary Fig. 7 (A) Heatmap of sample similarity clustering, (B) PCA and (C) Venn Diagram of differential genes between normal, ZIF-8/PEG200 and ZIF-8/PEG200@Mg group.

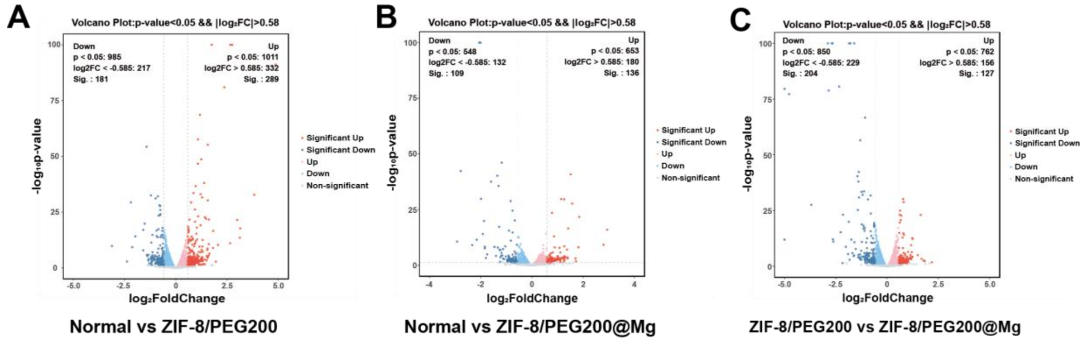

Supplementary Fig. 8 (A-C) Differential gene volcano mapping between normal, ZIF-8/PEG200 and ZIF-8/PEG200@Mg group.

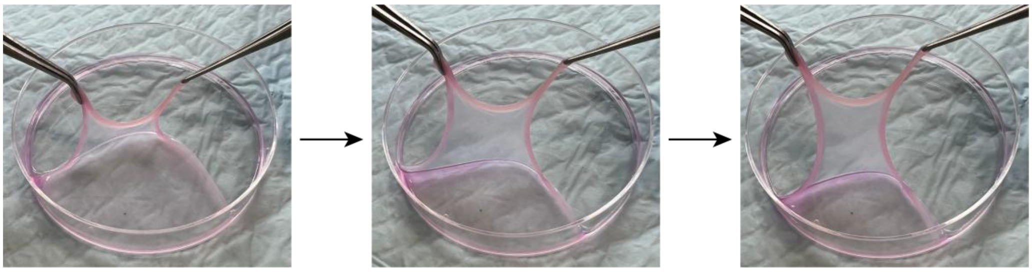

Supplementary Fig. 9 Pictures of the process of removing cell membrane sheets from the culture medium.

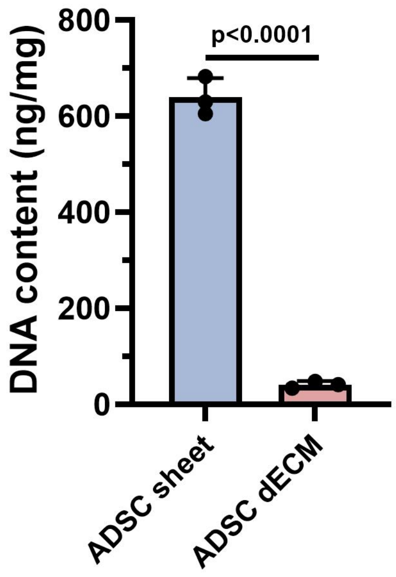

Supplementary Fig. 10 DNA contents of ADSCs sheet before/after decellularization. Data are expressed as mean ± SD (n = 3 biologically independent samples). p values calculated using one-tailed unpaired t-test. Source data are provided as a Source Data file.

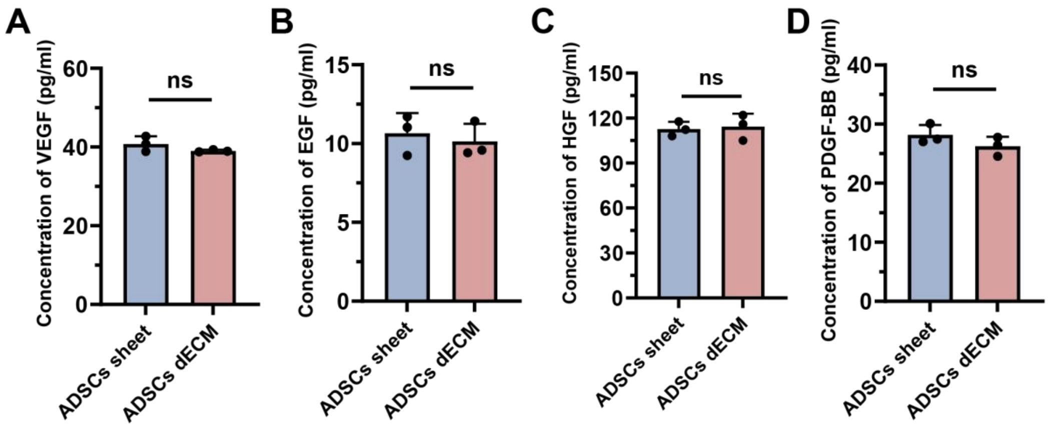

Supplementary Fig. 11 Concentrations of (A) VEGF, (B) EGF, (C) HGF, and (D) PDGF-BB in ADSCs sheets and ADSCs dECM, as determined by ELISA. Data are expressed as mean ± SD (n = 3 biologically independent samples). ns, not significant ($p > 0.05$). p values calculated using one-tailed unpaired t-test. Source data are provided as a Source Data file.

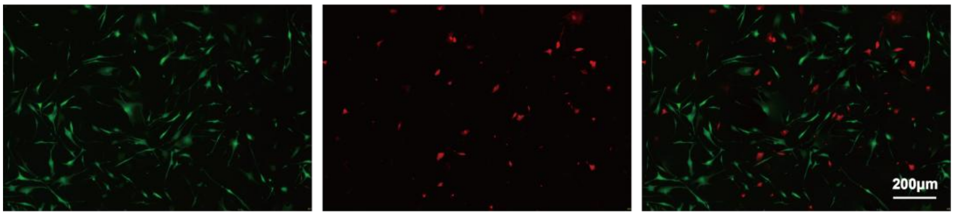

Supplementary Fig. 12 PI-positive photo to demonstrate that the PI staining was done correctly. n = 3 biologically independent samples.

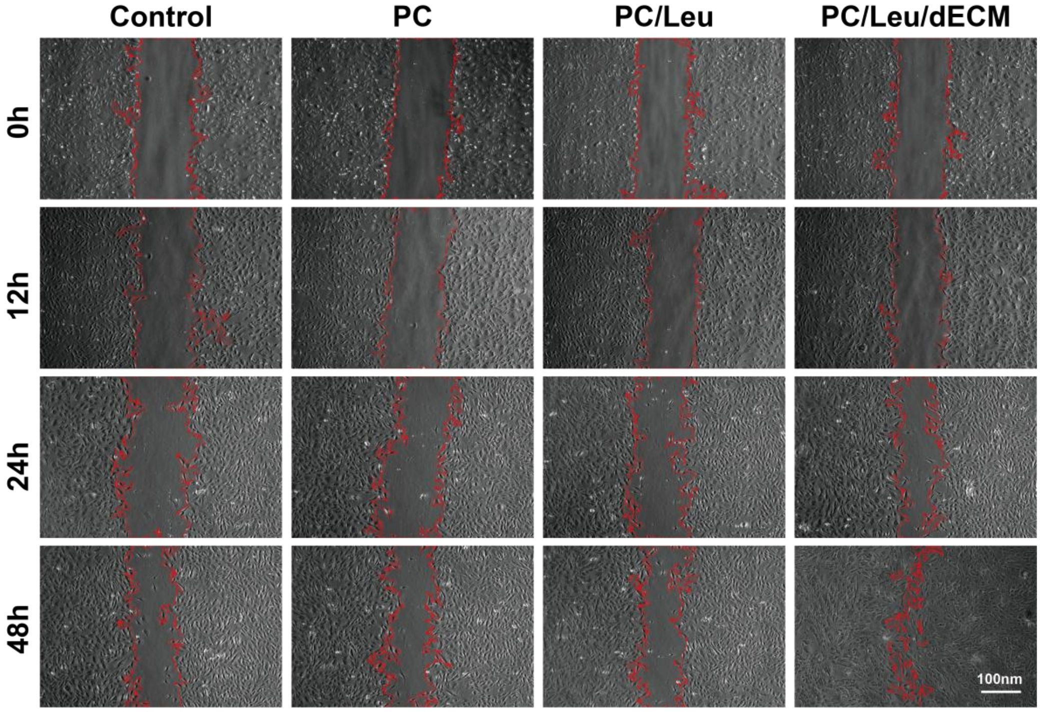

Supplementary Fig. 13 Bright-field image of ADSCs cultured under different conditions (PC, PC/Leu and PC/Leu/dECM) for 12h, 24h and 48h in wound healing experiments. n = 3 biologically independent samples.

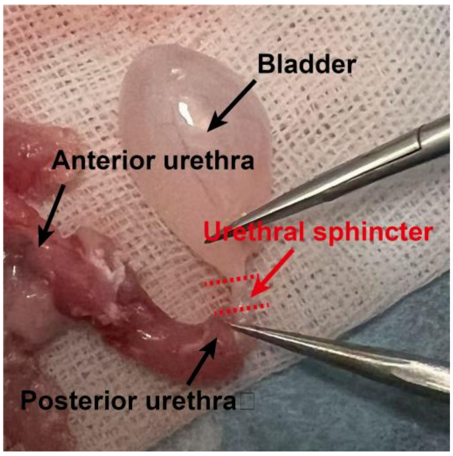

Supplementary Fig. 14 Schematic diagram of the anatomy of the urethral sphincter at the neck of the rat bladder.

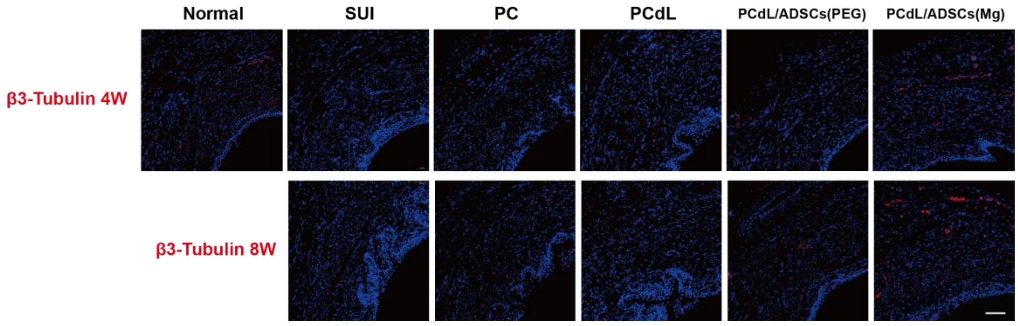

Supplementary Fig. 15 Immunofluorescence staining images of β3-Tubulin. Blue color represents the nucleus and red color represents β3-Tubulin. Scale bars: 100 μm. n = 3 biologically independent samples.

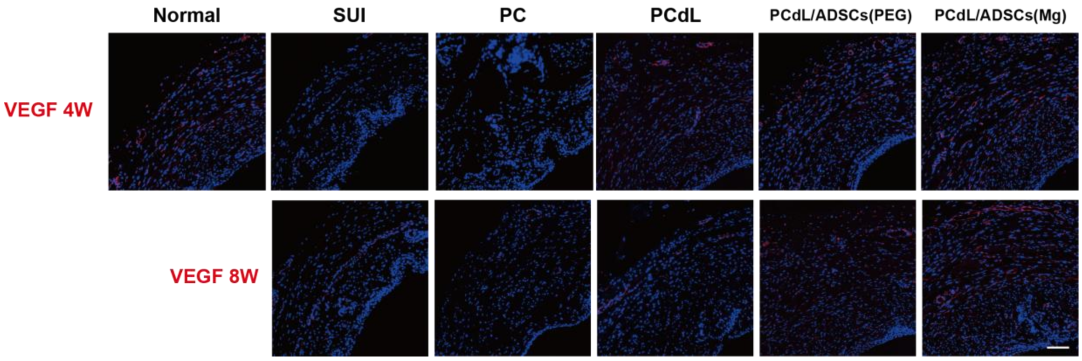

Supplementary Fig. 16 Immunofluorescence staining images of VEGF. Blue color represents the nucleus and red color represents VEGF. Scale bars: 100 μm. n = 3 biologically independent samples.

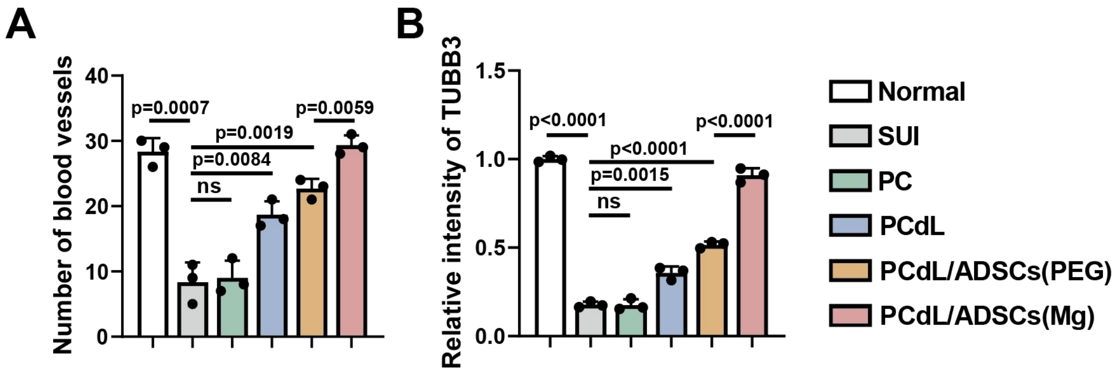

Supplementary Fig. 17 Number of blood vessels and relative fluorescence intensity of TUBB3 of urethral sphincter after 4 weeks of treatment with different injection systems. Data are expressed as the mean ± SD (n = 3 biologically independent samples). ns, not significant ($p > 0.05$). p values calculated using one-tailed unpaired t-test. Source data are provided as a Source Data file.

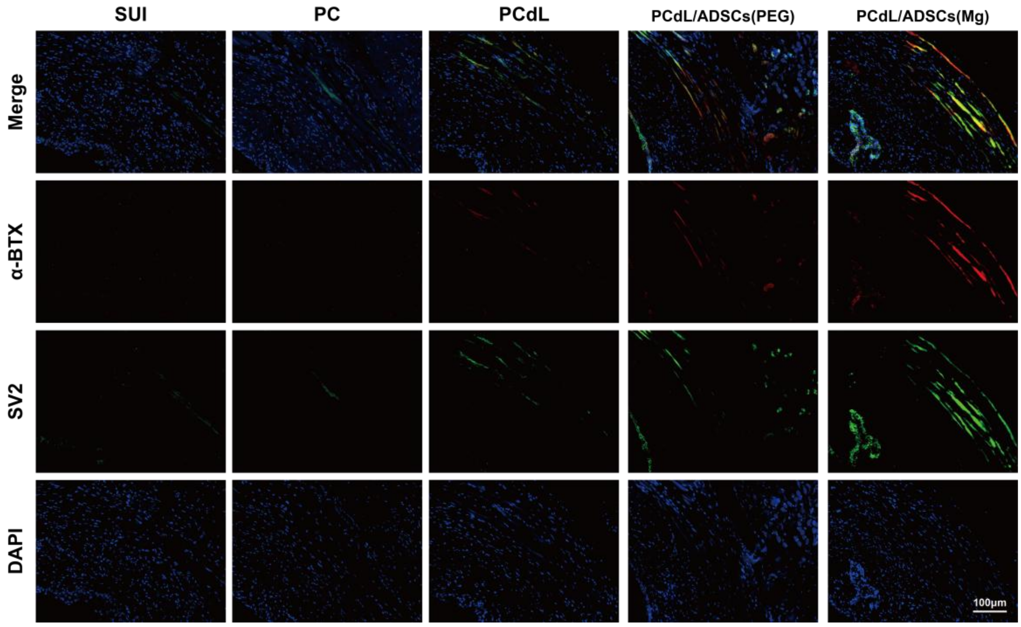

Supplementary Fig. 18 Representative pictures of NMJ identified by α-Bungarotoxin (α-BTX, postsynaptic) and SV2 (presynaptic). Scale bars: 100 μm. n = 3 biologically independent samples.

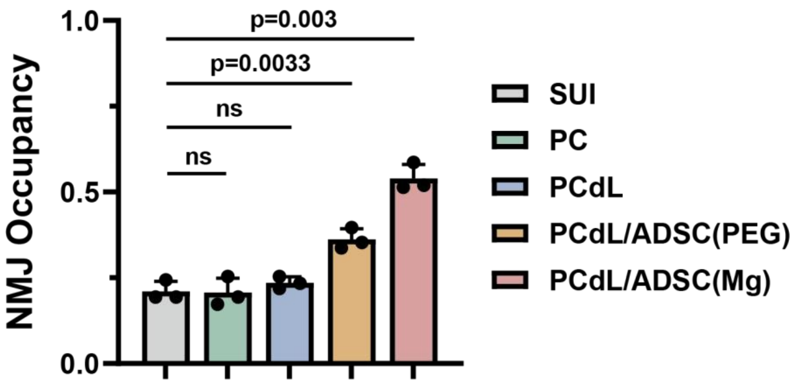

Supplementary Fig. 19 NMJ occupancy of local tissues after 8 weeks of treatment with different injection systems. Data are expressed as the mean ± SD (n = 3 biologically independent samples). ns, not significant ($p > 0.05$). p values calculated using one-tailed unpaired t-test. Source data are provided as a Source Data file.

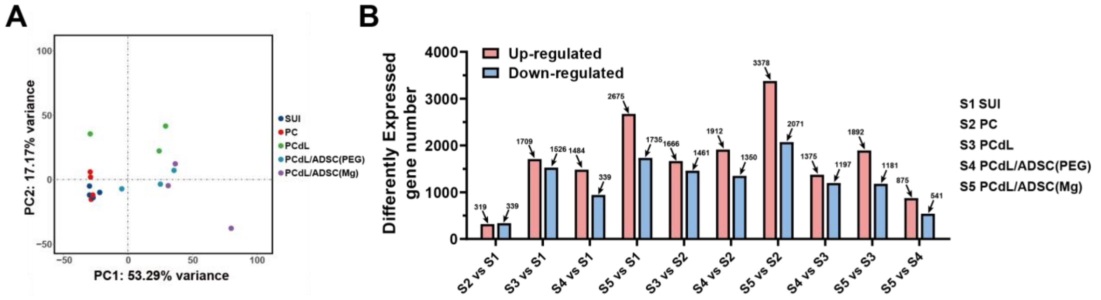

Supplementary Fig. 20 (A) PCA of all samples from different groups and (B) comparison of the number of differential genes ($p < 0.05$) between groups.

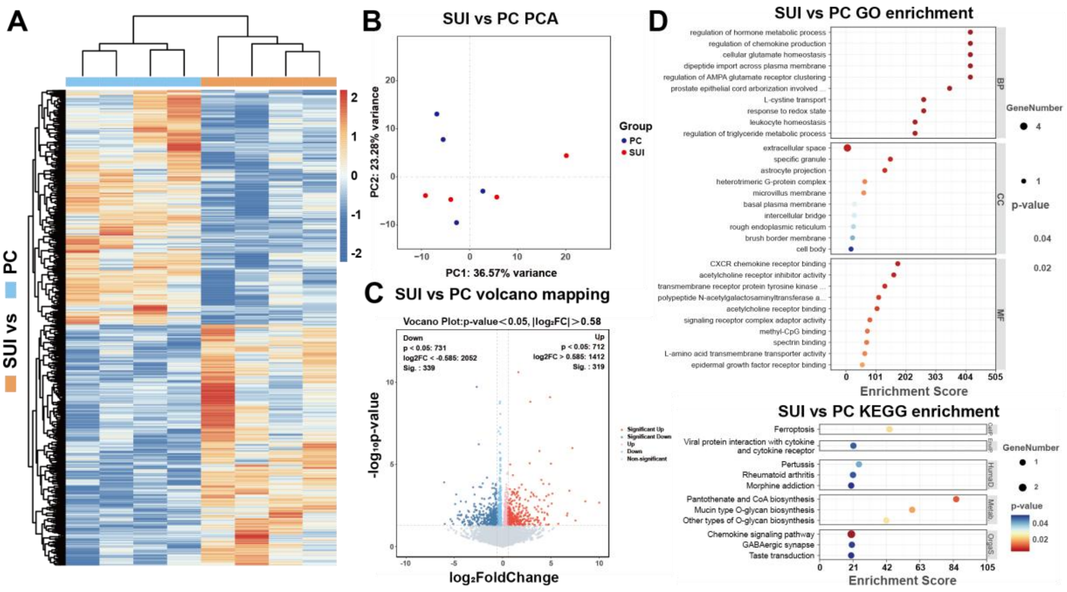

Supplementary Fig. 21 (A) Heat maps of urethral sphincter tissues between SUI and PC group which are used to evaluate the whole gene difference. (B) PCA analysis and (C) differential gene volcano mapping between SUI and PC group. (D) Up-regulated pathways in GO and KEGG enrichment analysis after RNA-sequencing between SUI and PC group.

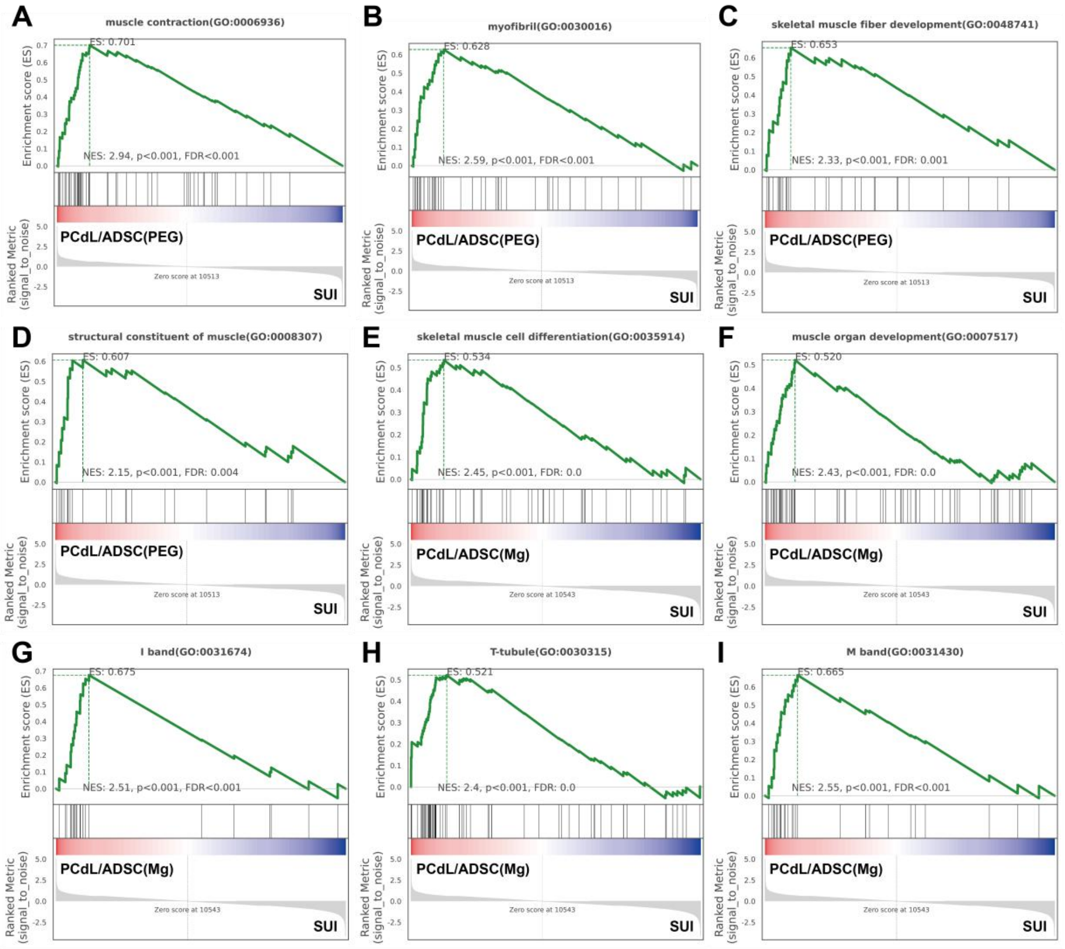

Supplementary Fig. 22 Gene Set Enrichment Analysis (GSEA) confirmed that the expression of pathways related to muscle regeneration and contraction were increased in the PCdL@ADSCs (PEG) and PCdL@ADSCs (Mg) group. (A) Muscle contraction. (B) Myofibril. (C) Skeletal muscle fiber development. (D) Structural constituent of muscle. (E) Skeletal muscle cell differentiation. (F) Muscle organ development. (G) I band. (H) T-tubule. (I) M band.

2. Supplementary tables

Supplementary Table 1. The forward and reverse primer sequences of different genes for qRT-PCR analysis.

Gene name	Forward primer	Reverse primer
GAPDH	CTGGAGAAACCTGCCAAGTATG	GGTGGAAGAATGGGAGTTGCT
Shank3	CCTGCCCTATCTGGAGTTTCG	GAACTTCTTCAGGTTTGCCTTTGT
Acta2	ACCATCGGGAATGAACGCTT	CTGTCAGCAATGCCTGGGTA
Clasp2	CTTCTTCCAGCATTTGCGTTTG	CAAAACCGTTGAAAGATGGG
Plxnb1	GCCCAGCCTACTAACAACCCTAA	AGGGTCATTTGCAGCCACATAC
Areg	CAGGATTACTTTGGCGAACGG	CGCAGCCTCCTTCTTTCTTCT
Ntn1	CTTGCATTAAGATTCCTGTAGCG	TTTGTCCGCCTTCAGAATGTG
Fgfbp3	CAACGGGCTGGACCAGAAT	GAATCCCGACTAGCCTACCC
Fez1	CTCCAGTGAAGAACCAGTTGCAG	CTCCTCGTTGATACCAGAGTCGTT
Nptxr	TCTGAGCCTGAAAGACAGCAACT	TGTGCAATGTCACCGACGAAG
Kirrel3	TCGTCAGCACACTCTTCATCTCC	TGGATGCTGGATGTCTATGGTG
Atxn7	CAAACCACAGGCATTTCAGTC	GGCAATCTGAGTAACTTGGAGC
Wasf2	CCATCTGTAGGATTCGGGTCT	ATCACTCACAGCAGGCAACGA
Itga2	ACAAACACCTTCAAGGCTATCCAAT	ATGCCAAACCTCAGTATCTCGT
ATP2A1	TACATGGCAATTGGGGGCTAC	CAGTCCAGGCCATCAAATTCAG
Mapt	AAAAAGGTGGCAGTGGTTCG	GTGTTTGATATTGTCCTTTGAGCC
Ache	TCGGGTCTATGCCTACATCTTTG	TTCAGGTTCAGGCTCACGTATT
Klhl41	GAGTGGCAATCCACAAAGGC	AGTGGGCGCGAACTCTTTAG
Smyd1	ATGCTGGTCACATTGAAGTAGGG	GAACATACGCAGCTCCATCTCT
Myoz1	CCCCTAACAAGAGGAGGAAGTCA	TGAGCTGTCAGAGAAAACATCGG
Myh1	AGTTGCATCCCTAAAGGCAGA	GGCTTGTTCTGAGCCTCGAT
Mef2c	AGGTAACACAGGTGGTCTGATGG	GGAGATCTGGCTTACGATTATTCA
Musk	GGAGCAAATCACAGAAAGAAAGC	AATGGGCATAGGAGTAGGTAGCA
Dok7	TACTGGGCTGGAGTCTTCTTCTTG	AGAGGAACTGGATAGACTTCGATCA
Ky	TGAATGAGCTGGTGAGTGACTTG	CTCAGGATGTTCGTGGGTTTG
Chrnd	GTTTCTGACTCGGGCCATGT	GGGGTAACTGCGGTTGTCTT

Supplementary Table 2. Antibody provider and catalog number.

Primary antibody	Provider	Catalog	Primary antibody	Provider	Catalog
GAPDH	abcam	ab8245	VEGF	HUABIO	ET1604-28
Acta2	HUABIO/abcam	ET1607-53/ab7817	TUBB3	abcam	ab18207
Fez1	abcam	Ab309358	Myoz1	proteintech	13160-1-AP
Atxn7	abcam	Ab259829	SERCA1	abcam	ab2818
Shank3	CST	cst14629T	Smyd1	HUABIO	ER1916-91
Clasp2	CST	cst64555T	Mef2c	abcam	ab211493
Desmin	HUABIO	ET1606-30	Myh1/2	abcam	ab37484
Synaptophysin	abcam	ab32127	Dok7	R&DSystems	AF6398-SP
AChE	HUABIO/abcam	ER62571/ab183591	Musk	R&DSystems	AF562-SP
Mapt	Affinity	AF3148			

Secondary antibody	Provider	Catalog
Rabbit Anti-Mouse IgG/HRP	Beijing Solarbio Science & Technology Co., Ltd.	K1031R-HRP
Goat Anti-Rabbit IgG H&L (Alexa Fluor® 647)	abcam	ab150083
Goat Anti-Rabbit IgG H&L (Alexa Fluor® 488)	abcam	ab150077
Goat Anti-Rabbit IgG H&L (HRP)	abcam	ab205718
Rabbit Anti-Goat IgG H&L (HRP)	abcam	ab6741

Supplementary Table 3. All abbreviations in the article and their specific meanings.

Abbreviation	Full name	Abbreviation	Full name
SUI	Stress urinary incontinence	ADSCs	Adipose-derived stem cells
ECM	Extracellular matrix	NMJ	Neuromuscular junction
t-BA	tert-butyl acrylate	NIPAm	N-isopropylacrylamide
PNIPAm-C	Carboxyl-modified poly-NIPAm	dECM	decellularized ECM
EDC	1-ethyl-3-[3-dimethylaminopropyl] carbodiimide hydrochloride	NHS	N-hydroxysuccinimide
PCdL	PNIPAm-C/leucine/dECM	ZIF-8	Zeolitic imidazolate framework-8
MOFs	Metal-organic frameworks	Mg^{2+}	Magnesium ions
NPs	Nanoparticles	LPP	Leak point pressure
qRT-PCR	Quantitative reverse transcription polymerase chain reaction	WB	Western blot
Leu	Leucine	PBS	Phosphate Buffered Saline
2-MIM	2-methylimidazole	PEG	Polyethylene glycol
$MgCl_2$	Magnesium chloride	RNA	Ribonucleic acid
a.u.	Arbitrary unit	DEGs	Differentially expressed genes
CCK8	Cell Counting Kit 8	KEGG	Kyoto Encyclopedia of Genes and Genomes
GO	Gene Ontology	HUVECs	Human umbilical vein endothelial cells
VEGF	Vascular endothelial growth factor	PDGF-BB	Platelet-derived growth factor-BB
EGF	Epidermal growth factor	HGF	Hepatocyte growth factor
PC	PNIPAm-C polymer	PC/Leu	PNIPAm-C/leucine polymer
PC/Leu/dECM	PNIPAm-C/leucine/ADSCs sheets-derived dECM powder polymer	PI	Propidium Iodide
3D	Three dimension	SD	Standard deviation

HE	Hematoxylin-eosin staining	α-SMA	α-smooth muscle actin
TUBB3	tubulin β3	α-BTX	α-bungarotoxin
PCdL/ADSCs (Mg)	PCdL+ADSCs+ZIF-8/PEG200 @Mg	PCdL/ADSCs (PEG)	PCdL+ADSCs+ZIF-8/PEG200
MUSK	Skeletal receptor tyrosine-protein kinase	CM	Congenital myasthenia
DOK7	Docking Protein 7		
