# Supplementary material for: Injectable therapeutic system incorporating neurogenesis-programmed stem cells concomitantly promoting muscle regeneration treats stress urinary incontinence
Source: Nat Commun. 2025 Sep 25;16:8404. doi: 10.1038/s41467-025-63421-2 (PMC12462450; doi:10.1038/s41467-025-63421-2)
Supplement: Supplementary file 2 — Description of Additional Supplementary Files [file 41467_2025_63421_MOESM2_ESM.pdf]

Title: Supp. Movie 1

Description: Video of detaching ADSCs cell sheets from the culture dish.
