# Supplementary material for: Injectable therapeutic system incorporating neurogenesis-programmed stem cells concomitantly promoting muscle regeneration treats stress urinary incontinence
Source: Nat Commun. 2025 Sep 25;16:8404. doi: 10.1038/s41467-025-63421-2 (PMC12462450; doi:10.1038/s41467-025-63421-2)
Supplement: Supplementary file 6 — Source Data [file 41467_2025_63421_MOESM6_ESM.zip › Source Data/Supplementary files (WB scans).pdf]

Figure 3F

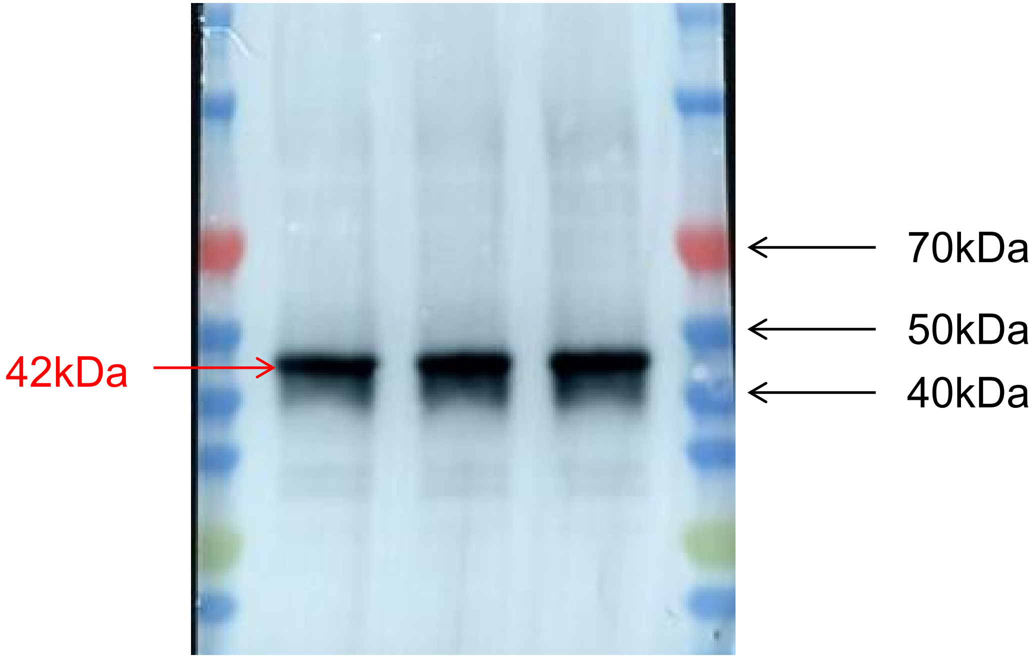

Acta2

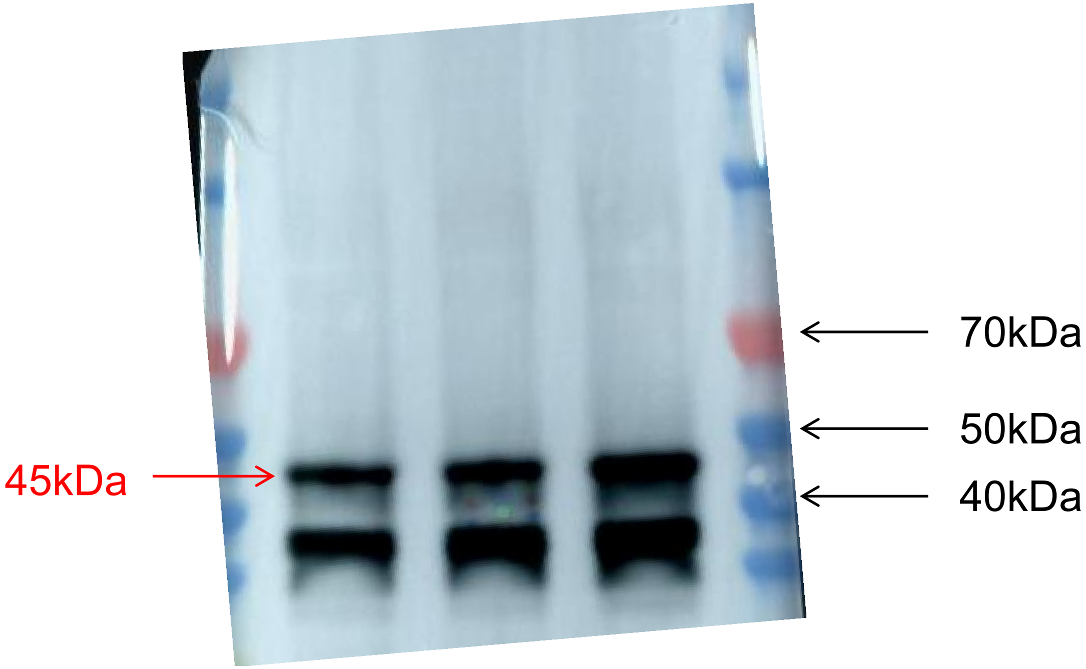

Fez1

Figure 3F

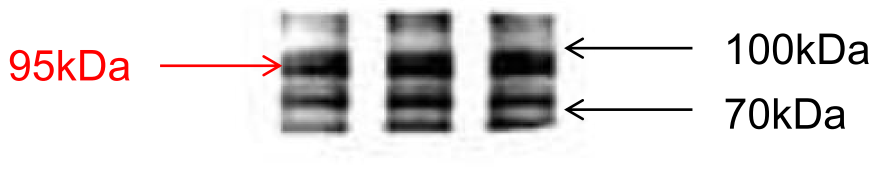

Atxn7

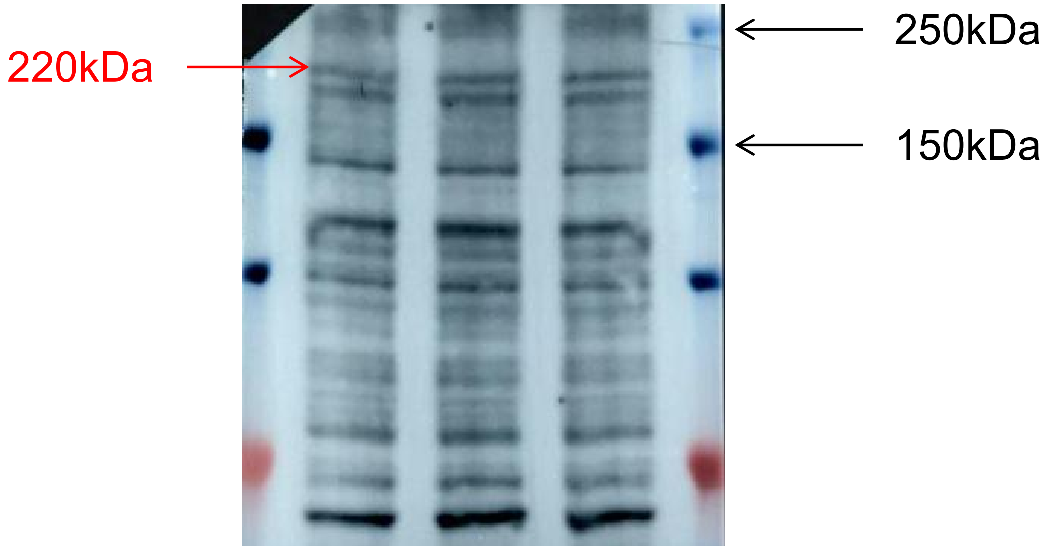

Shank3

Figure 3F

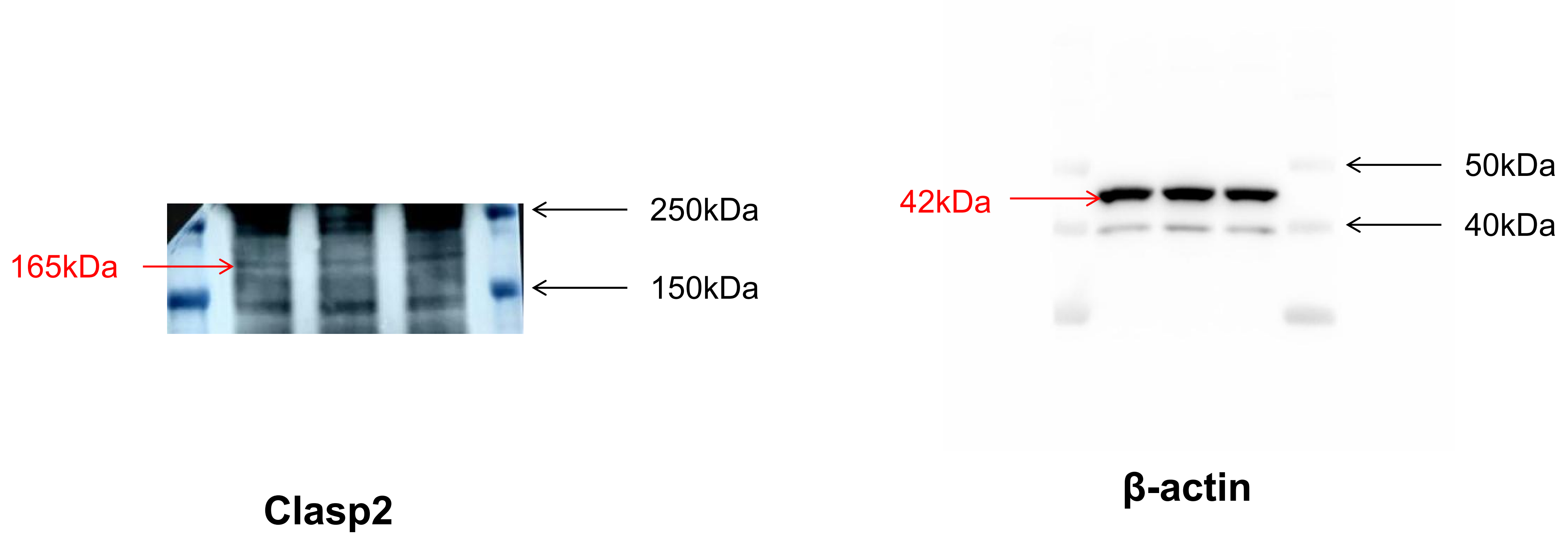

Figure 8F

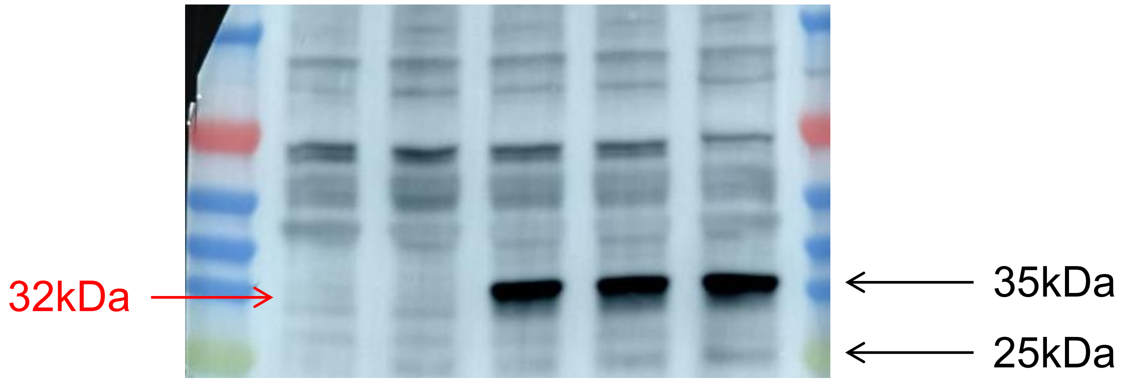

Myoz1

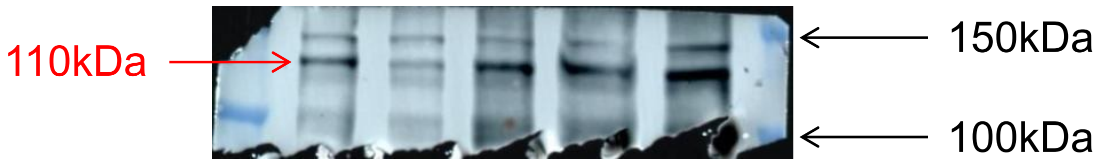

SERCA1

Figure 8F

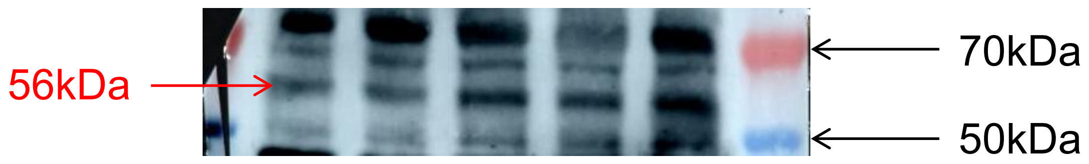

Smyd1

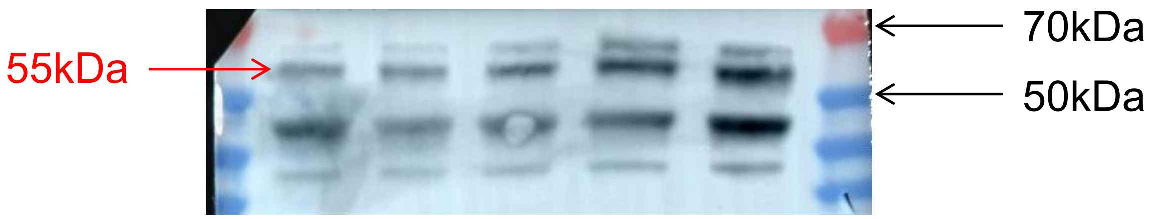

Mef2c

Figure 8F

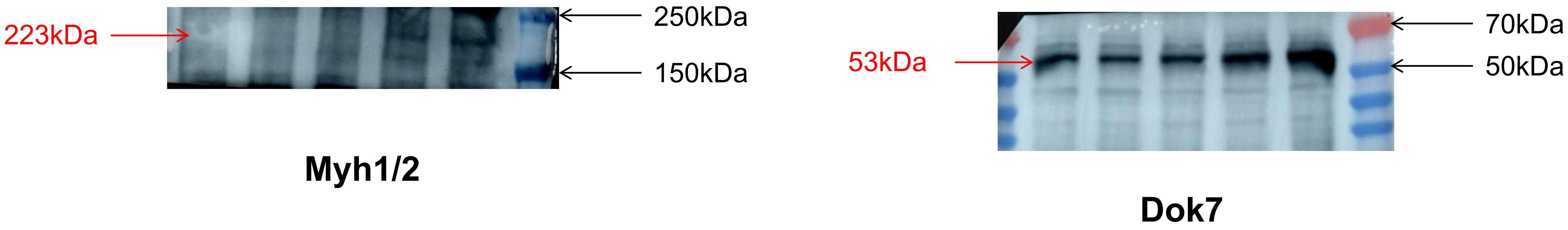

Figure 8F

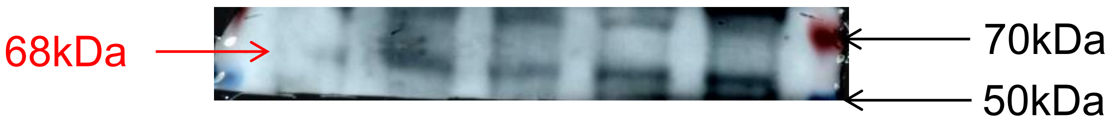

Ache

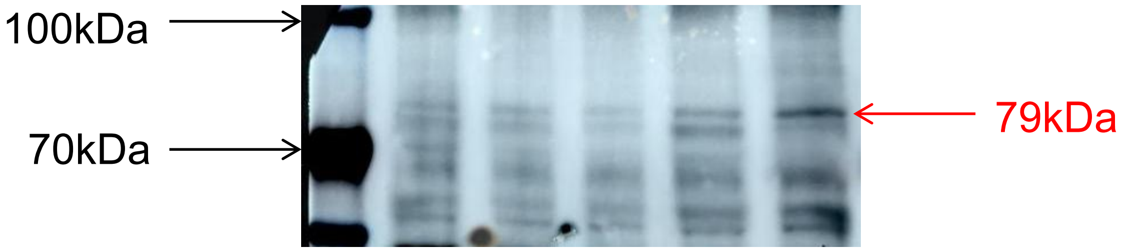

Mapt

Figure 8F

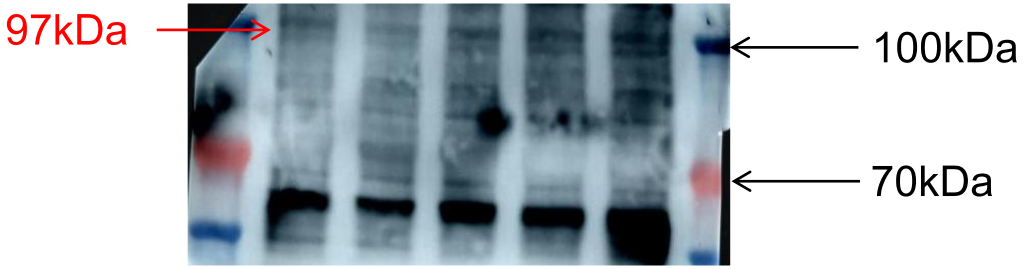

Musk

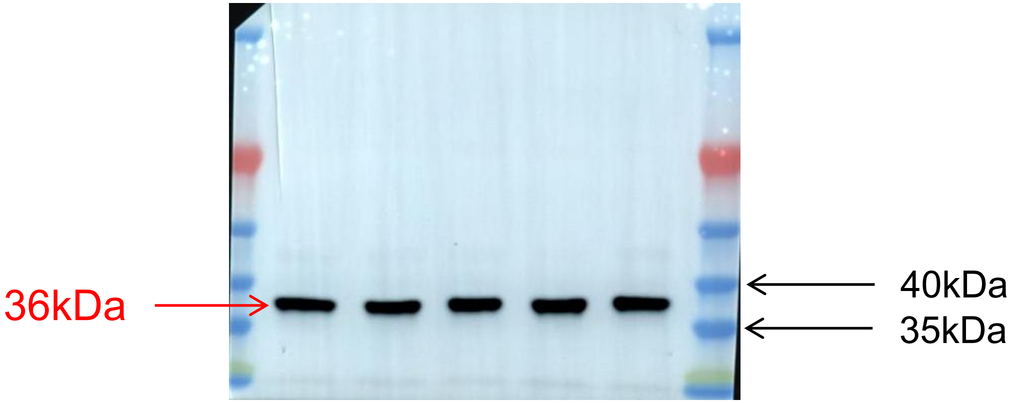

GAPDH
