# Supplementary material for: Injectable therapeutic system incorporating neurogenesis-programmed stem cells concomitantly promoting muscle regeneration treats stress urinary incontinence
Source: Nat Commun. 2025 Sep 25;16:8404. doi: 10.1038/s41467-025-63421-2 (PMC12462450; doi:10.1038/s41467-025-63421-2)
Supplement: Supplementary file 6 — Source Data [file 41467_2025_63421_MOESM6_ESM.zip › Source Data/Supplementary files (WB scans).pptx]

## Slide 1
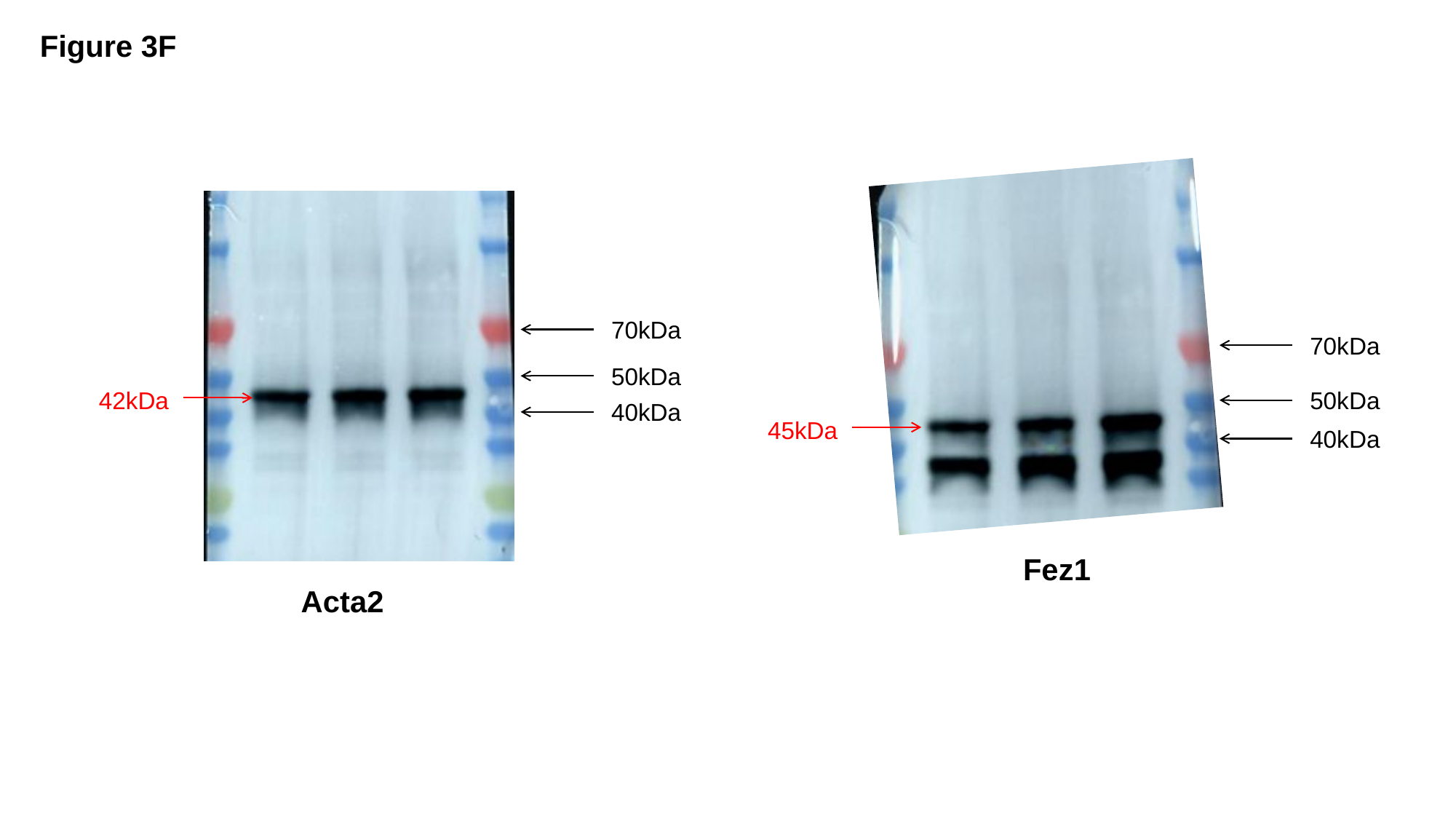

Figure 3F
70kDa
70kDa
50kDa
42kDa
50kDa
40kDa
45kDa
40kDa
Fez1
Acta2

## Slide 2
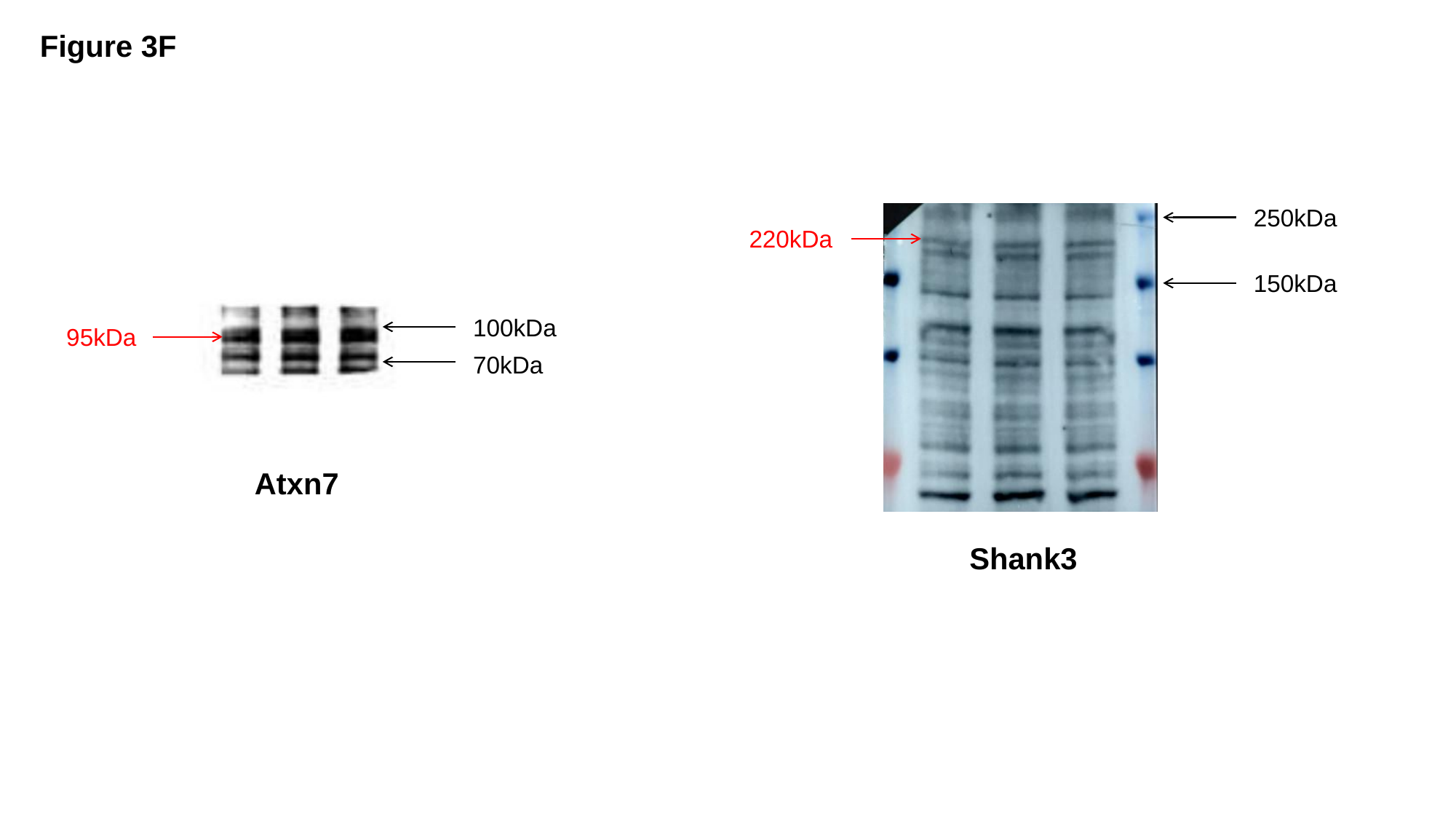

Figure 3F
250kDa
220kDa
150kDa
100kDa
95kDa
70kDa
Atxn7
Shank3

## Slide 3
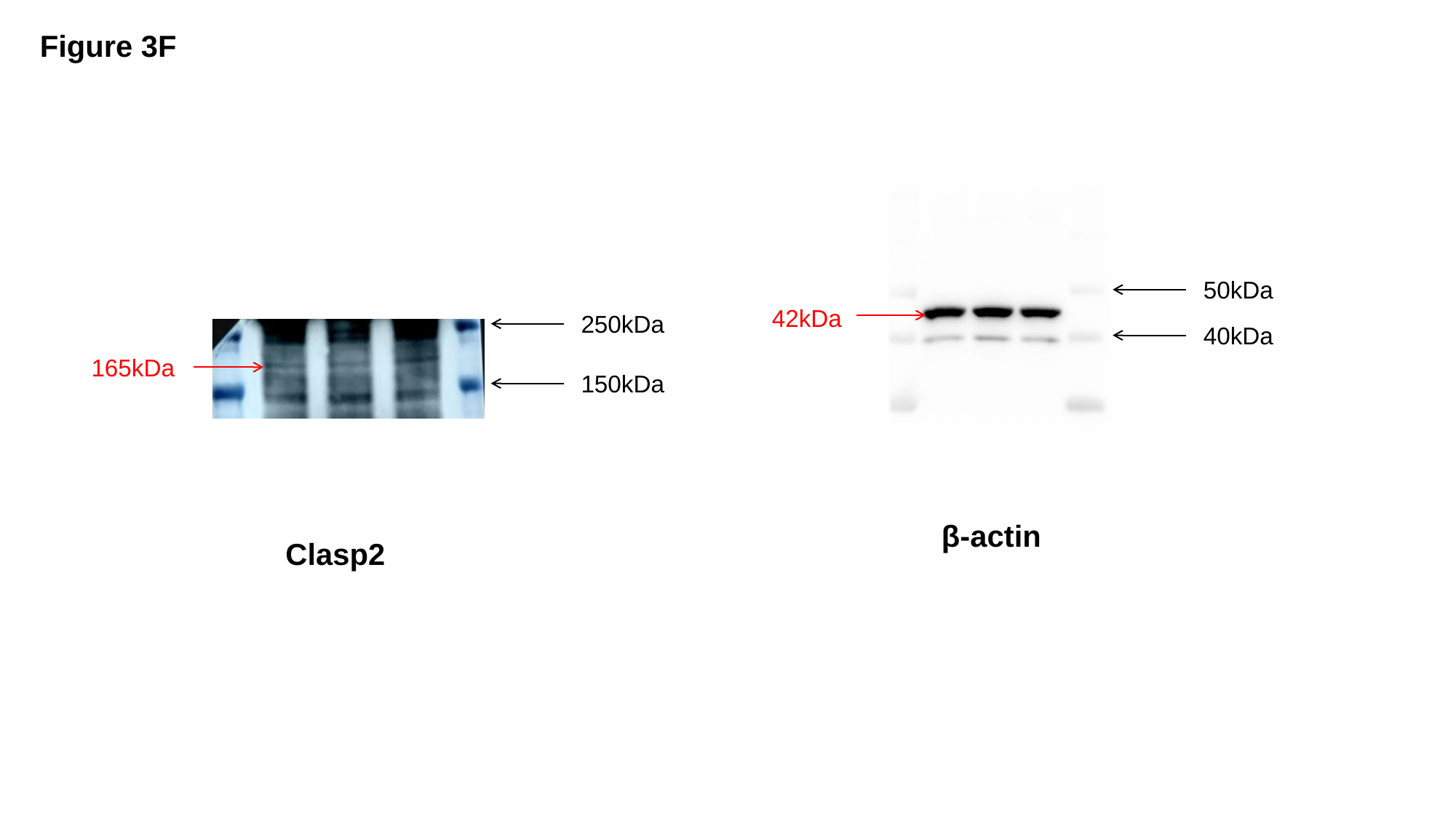

Figure 3F
50kDa
42kDa
250kDa
40kDa
165kDa
150kDa
β-actin
Clasp2

## Slide 4
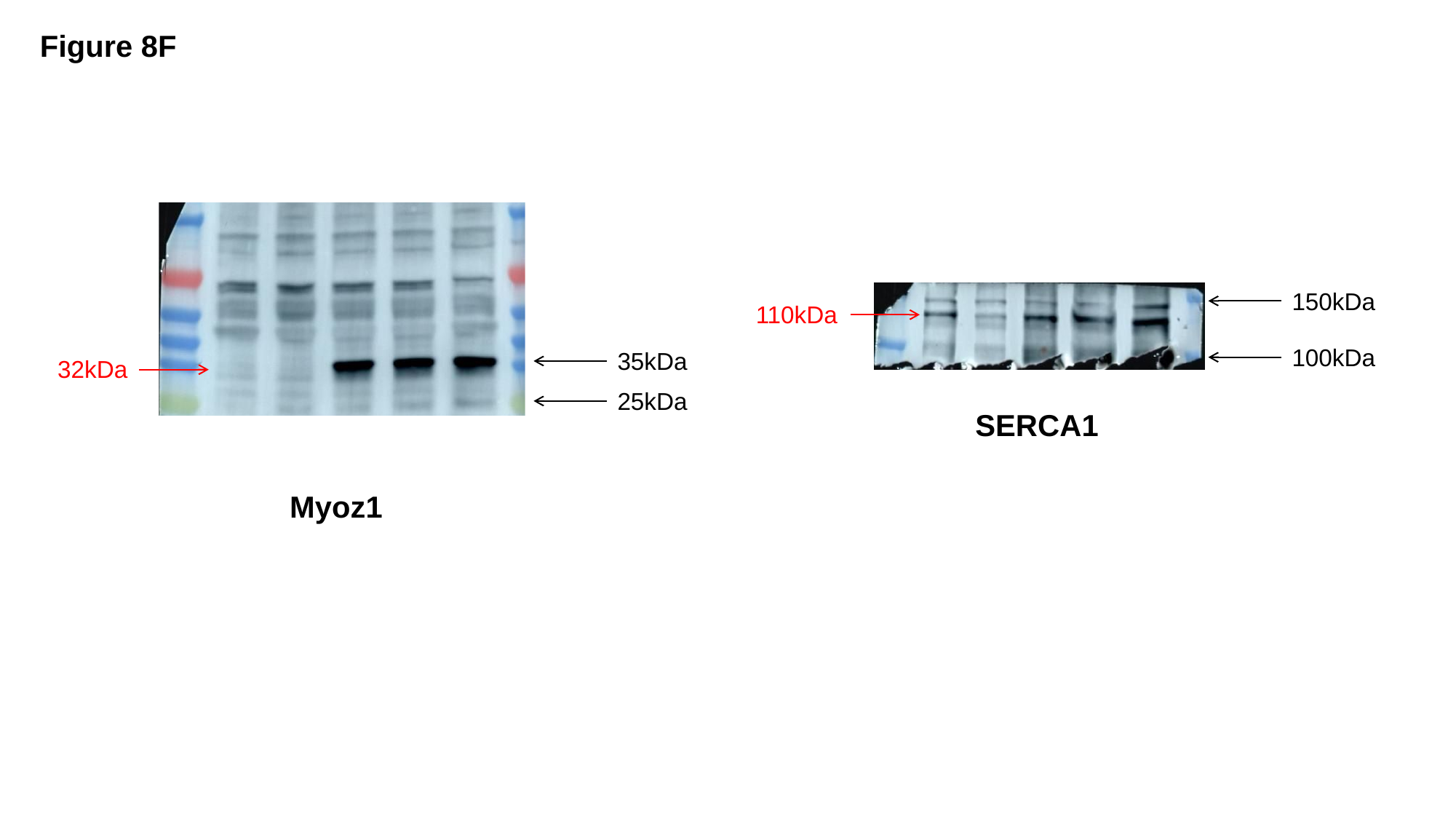

Figure 8F
150kDa
110kDa
100kDa
35kDa
32kDa
25kDa
SERCA1
Myoz1

## Slide 5
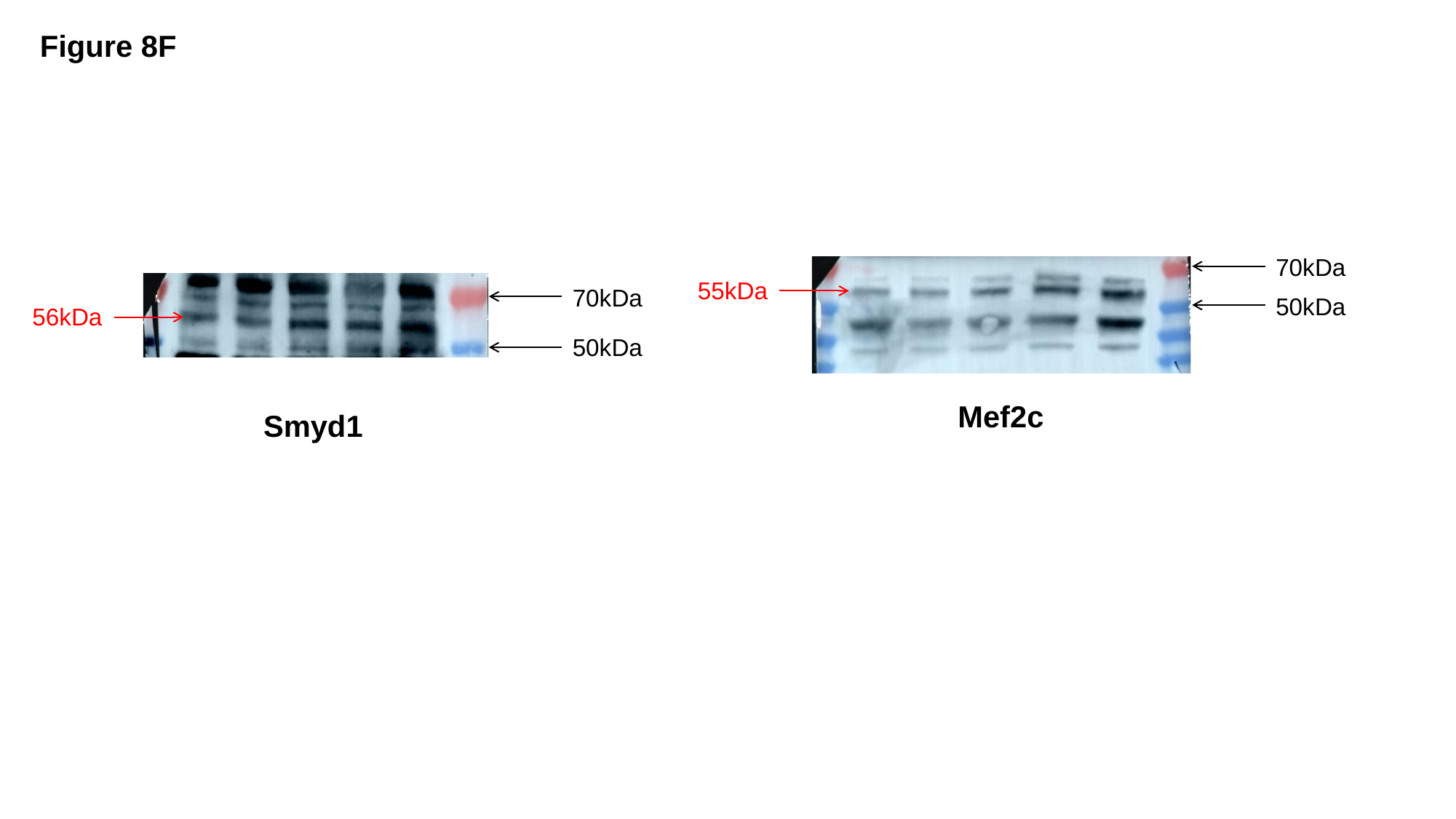

Figure 8F
70kDa
55kDa
70kDa
50kDa
56kDa
50kDa
Mef2c
Smyd1

## Slide 6
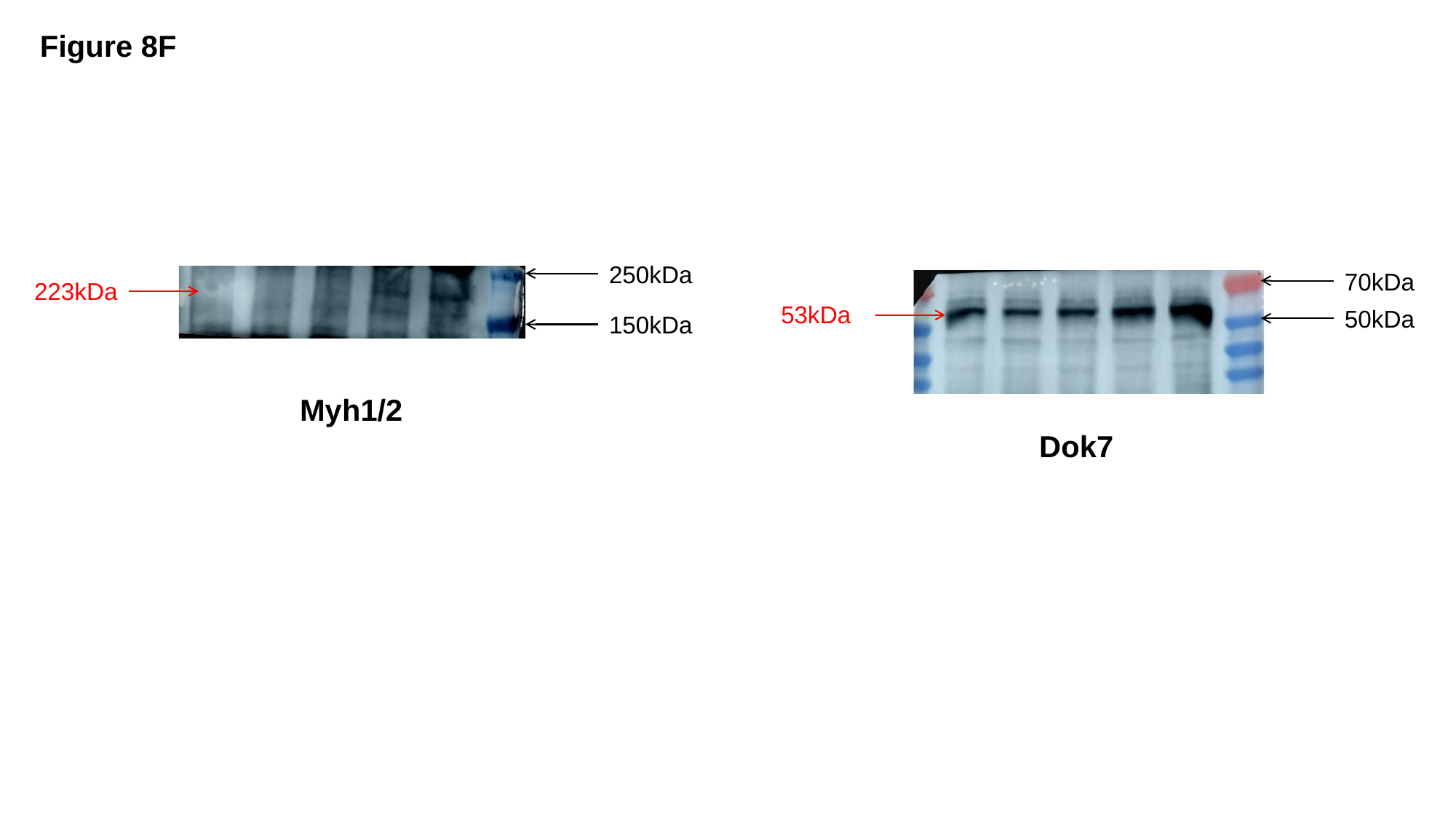

Figure 8F
250kDa
70kDa
223kDa
53kDa
50kDa
150kDa
Myh1/2
Dok7

## Slide 7
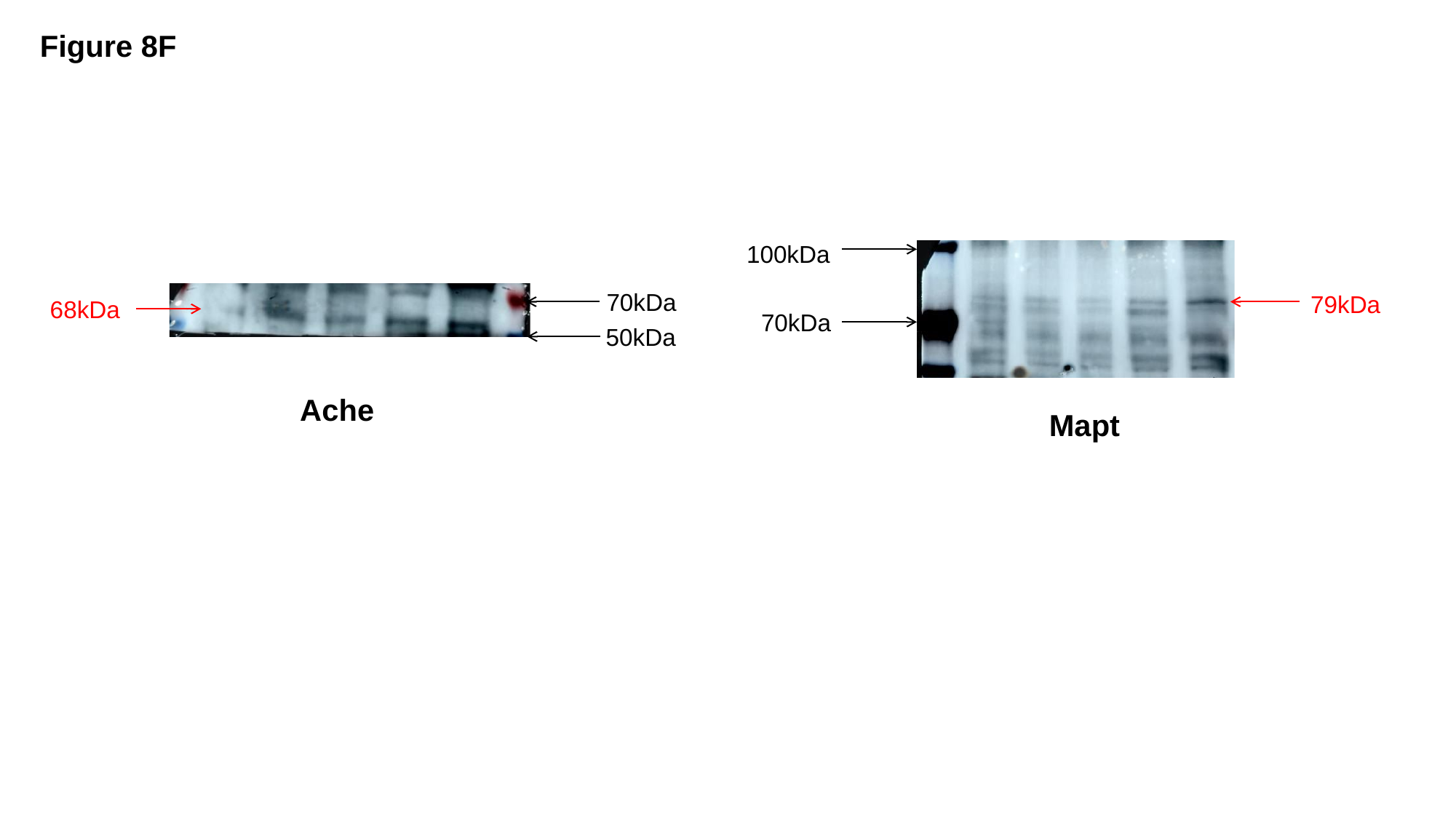

Figure 8F
100kDa
70kDa
79kDa
68kDa
70kDa
50kDa
Ache
Mapt

## Slide 8
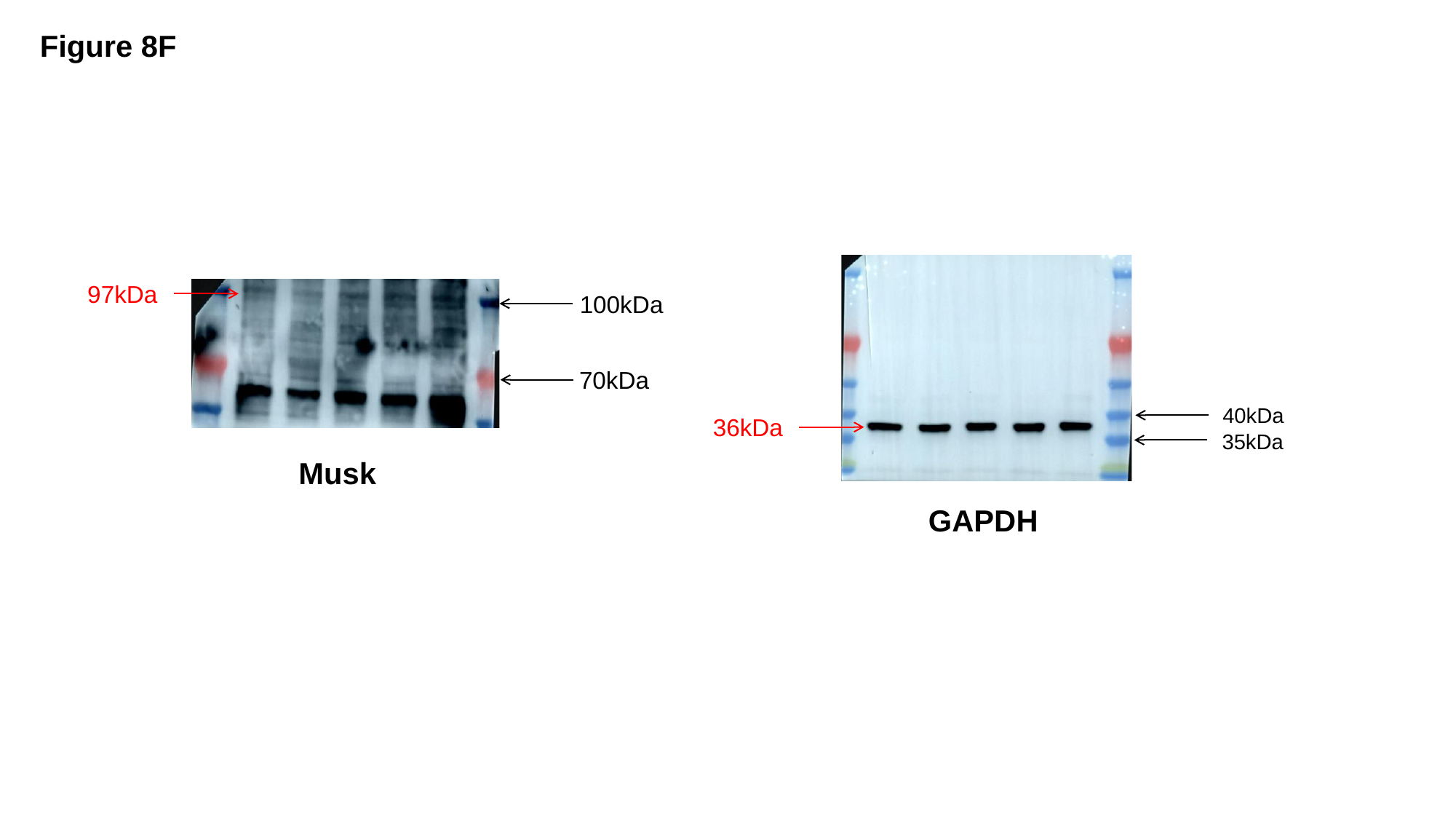

Figure 8F
97kDa
100kDa
70kDa
40kDa
36kDa
35kDa
Musk
GAPDH
