# Supplementary figures and images for: Injectable therapeutic system incorporating neurogenesis-programmed stem cells concomitantly promoting muscle regeneration treats stress urinary incontinence
Source: Nat Commun. 2025 Sep 25;16:8404. doi: 10.1038/s41467-025-63421-2 (PMC12462450; doi:10.1038/s41467-025-63421-2)

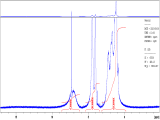

Supplement: Supplementary file 6 — Source Data [file 41467_2025_63421_MOESM6_ESM.zip › Source Data/Source Data (1H NMR)/PNIPAm/pdata/1/thumb.png]

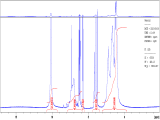

Supplement: Supplementary file 6 — Source Data [file 41467_2025_63421_MOESM6_ESM.zip › Source Data/Source Data (1H NMR)/PNIPAm-C/pdata/1/thumb.png]
